# An enhancement algorithm for head characteristics of caged chickens detection based on cyclic consistent migration neural network

**DOI:** 10.1016/j.psj.2024.103663

**Published:** 2024-03-15

**Authors:** Zhenwei Yu, Liqing Wan, Khurram Yousaf, Hai Lin, Ji Zhang, Hongchao Jiao, Geqi Yan, Zhanhua Song, Fuyang Tian

**Affiliations:** ⁎ College of Mechanical and Electronic Engineering, Shandong Agricultural University, Tai'an 271018, China; † Shandong Provincial Engineering Laboratory of Agricultural Equipment Intelligence, Shandong Provincial Key Laboratory of Horticultural Machineries and Equipment, Tai'an 271018, China; ‡ Atta-ur-Rahman School of Applied Biosciences, National University of Sciences and Technology, Islamabad 44000, Pakistan; § College of Animal Science and Technology, Shandong Agriculture University, Tai'an 271018, China

**Keywords:** cage door, caged chicken, object detection, cyclic consistent migration neural network

## Abstract

The enclosed multistory poultry housing is a type of poultry enclosure widely used in industrial caged chicken breeding. Accurate identification and detection of the comb and eyes of caged chickens in poultry farms using this type of enclosure can enhance managers’ understanding of the health of caged chickens. However, the accuracy of image detection of caged chickens will be affected by the enclosure's entrance, which will reduce the precision. Therefore, this paper proposes a cage-gate removal algorithm based on big data and deep learning Cyclic Consistent Migration Neural Network (CCMNN). The method achieves automatic elimination and restoration of some key information in the image through the CCMNN network. The Structural Similarity Index Measure (SSIM) between the recovered and original images on the test set is 91.14%. Peak signal-to-noise ratio (**PSNR**) is 25.34dB. To verify the practicability of the proposed method, the performance of the target detection algorithm is analyzed both before and after applying the CCMNN network in detecting the combs and eyes of caged chickens. Different YOLOv8 detection algorithms, including YOLOv8s, YOLOv8n, YOLOv8m, and YOLOv8x, were used to verify the algorithm proposed in this paper. The experimental results demonstrate that compared to images without CCMNN processing, the precision of comb detection of caged chickens is improved by 11, 11.3, 12.8, and 10.2%. Similarly, the precision of eye detection for caged chickens is improved by 2.4, 10.2, 6.8, and 9%. Therefore, more complete outline images of caged chickens can be obtained using this algorithm and the precision in detecting the comb and eyes of caged chickens can be enhanced. These advancements in the algorithm offer valuable insights for future poultry researchers aiming to deploy enhanced detection equipment, thereby contributing to the accurate assessment of poultry production and farm conditions.

## INTRODUCTION

Poultry farming has consistently held a vital role in agricultural production (Li et al., 2022). To accommodate the growing global population, the livestock industry needs to enhance its efficiency in delivering a greater supply of livestock products, all the while addressing concerns related to animal welfare, environmental sustainability, and public health (Pereira et al., 2020; Ren et al., 2020; Neethirajan and Kemp, 2021). Achieving real-time automated poultry detection stands as a fundamental requirement for precise poultry breeding (Ojo et al., 2022; Yang et al., 2022). The automation of detection, enumeration, and tracking of both individual birds and groups within the poultry sector is pivotal for elevating agricultural productivity and enhancing animal well-being (Okinda, et al., 2019; Astill et al., 2020; Neethirajan, 2022). Nyalala, et al. (2021) Owing to its non-invasive and non-intrusive nature and its capacity to present a wide range of information, computer vision systems can be applied in size, mass, volume determination, sorting and grading of poultry products.

With the development of science and technology, an increasing number of researchers are leveraging computer vision technology in the field of poultry breeding, leading to significant advancements (Wu et al., 2022). Such as, Guo et al. (2022) developed a convolutional neural network models (**CNN**) to monitor chicken behaviors (i.e., feeding, drinking, standing, and resting). Chen et al. (2023) proposed an approach based on deep learning to develop an automatic warning system for anomalous dispersion and movement of chicken flocks in commercial chicken farms. Guo et al. (2023b) established a laying hens detection model called YOLOv5-C3CBAM-BiFPN, and tested its performance in detecting birds on open litter. Subedi et al. (2023) developed, trained, and compared 3 new deep learning models, which are: YOLOv5s-egg, YOLOv5x-egg, and YOLOv7-egg networks, in tracking floor eggs in 4 research cage-free laying hen facilities. Ma et al. (2022) proposed a chicken face detection network with an augmentation module. To improve the accuracy and efficiency of broiler stunned state recognition, Ye et al. (2020a) proposed an improved fast region-based convolutional neural network (You Only Look Once + Multilayer Residual Module (YOLO + MRM)) algorithm and applied to the recognition of 3 broiler stunned states: insufficient, appropriate and excessive stuns. Fang et al. (2022) designed a poultry pose-estimation system, which realized the automatic pose estimation of a single broiler chicken using a multi-part detection method. Fang et al. (2020) demonstrate the use of a deep regression network to track single poultry based on computer vision technology. Vroegindeweij et al. (2018) proposed a simple and robust method for image pixel classification based on spectral reflectance properties. Del Valle et al. (2021) used the restlessness index and computer vision technology to automatically assess the thermal comfort of poultry, effectively demonstrating the difference in poultry agitation under different heat stress conditions.

Concurrently, Geffen et al., (2020) spearheaded the development of a machine vision system engineered to automatically tally the number of hens within battery cages. Ye et al. (2020b) proposed an improved fast region-based convolutional neural network (**RCNN**) algorithm to enhance the accuracy and efficiency of recognizing broilers in a stunned state. Xiao et al. (2019) presented an automatic behavior information monitoring method for caged chickens using the binocular vision system. Nasirahmadi, et al. (2020) developed and tested a novel pecking activity detection tool for potential use on turkey farms by means of acoustic data and a Convolutional Neural Networks (**CNN**) Model. Wang, et al. (2022) developed a discrete model that incorporates Time-Period Groups (**TPG**), the group buffered rolling (**GBR**) mechanism, and TPG factors. Experimental results showed that it is a helpful tool for predicting indoor air temperatures and may help in the development of improved control strategies for the indoor thermal environment. Neethirajan (2022) introduced a deep learning model grounded in the You Only Look Once (**YOLOv5**) framework. This model demonstrates remarkable competence in discerning domesticated chickens within the videos characterized by intricate and diverse backgrounds. Zhuang and Zhang (2019) proposed a model structure to identify sick broilers within a flock by using digital image processing and deep learning, and the results indicate that sick broilers within a flock can be successfully detected automatically and the method has a potential to facilitate efficient flock management.

The comb and eyes of chickens are not only distinctive markers for differentiating breeds and genetic traits but also crucial indicators reflecting the overall health status and productivity of these avian species. Notably, a significant negative correlation was identified between laying age and both crown height and crown length (*P* < 0.01). While, an equally significant positive correlation existed between egg production and crown length as well as crown height (*P* < 0.01) (GUO et al., 2023a). Simultaneously, the comb and eyes serve as reference points for the selection of body weight and dimensions, underscoring the paramount importance of accurately detecting the comb and eyes of caged chickens. However, the presence of cage doors may introduce a spectrum of challenges, potentially impeding the performance of target detection algorithms.

Despite recent progress of object category detection in real scenes, detecting objects that are partially or heavily occluded remains a challenging problem due to the uncertainty and diversity of occlusion situations which could cause large intra-category appearance variance (Zhou and Yuan, 2020). At the same time, more and more researchers realize the influence of complex environment on the acquisition of target information, and begin to try to use image enhancement algorithms to obtain more complete target information. Tang et al. (2023) presented an enhanced version of their previous work, Pest-YOLO, replace the original squeeze-excitation attention mechanism with the ECA mechanism, effectively improving the model's ability to extract essential features from pest images. Sun et al. (2022) proposed an image enhancement approach for fish detection in complex underwater environment; the method first uses a Siamese network to obtain a saliency map and then multiplies this saliency map by the input image to construct an image enhancement module. Applying this module to the existing mainstream one-stage and two-stage target detection frameworks can significantly improve their detection accuracy. Gupta et al. (2020) presented an automated and effective technique for fence removal and image reconstruction using conditional Generative Adversarial Networks (**cGAN**). These networks have been successfully applied in several other domains of computer vision, focusing on image generation and rendering. Yang et al. (2022) proposed CCD (caged chicken defensing), a defensing algorithm based on U-Net and pix2pixHD, to improve caged chickens’ detection accuracy. The proposed defensing algorithm can accurately identify the cage wire mesh and recover the chicken contours completely. In the test set, the detection accuracy of the cage wire mesh was 94.71%, while a structural similarity (**SSIM**) of 90.04% and a peak signal-to-noise ratio (**PSNR**) of 25.24 dB were obtained in the image recovery.

Numerous researchers have extensively studied the challenge of the problem of occlusion removal. They proposed many methods to eliminate the occlusion in the images. However, once their proposed methods eliminated occlusions within images, the resultant new images frequently exhibit characteristics like reduced contrast, underexposure, diminished structural similarity compared to the original, and a decreased peak signal-to-noise ratio. Moreover, the methods used had problems such as resource-intensive processes, challenges in fine-tuning hyper-parameters, and limited capacity to address intricate scenes.

Therefore, this paper proposes a method for automatically removing chicken coops based on CCMNN network and deep learning. In this method, CCMNN network is employed to automatically remove the network cable obstructing the cage door in the image and recover the information of the chickens situated behind the cage door. Through this algorithm, a more comprehensive outline image of caged chickens can be acquired; especially focusing on the information of combs and eyes, and the accuracy of the detection algorithm for identifying combs and eyes of caged chickens can be enhanced. The proposed algorithm can significantly enhance the detection effectiveness of combs and eyes in caged chickens and serve as a reference for subsequent poultry researchers deploying detection equipment. This aids in achieving accurate poultry production and farm detection. Through the introduction of this automatic cage door removal technology, it is expected to achieve efficient, accurate, and non-destructive testing of combs and eyes in caged chickens, and make positive contributions to intelligent upgrading and sustainable development in the agricultural breeding industry. At the same time, the promotion and application of this technology will also strongly promote scientific and technological innovation within the poultry-farming field, and usher in new opportunities for enhancing chicken welfare, optimizing production processes, and improving product quality.

## MATERIALS AND METHODS

### Materials

#### Experimental Platform

**Figure 1:**
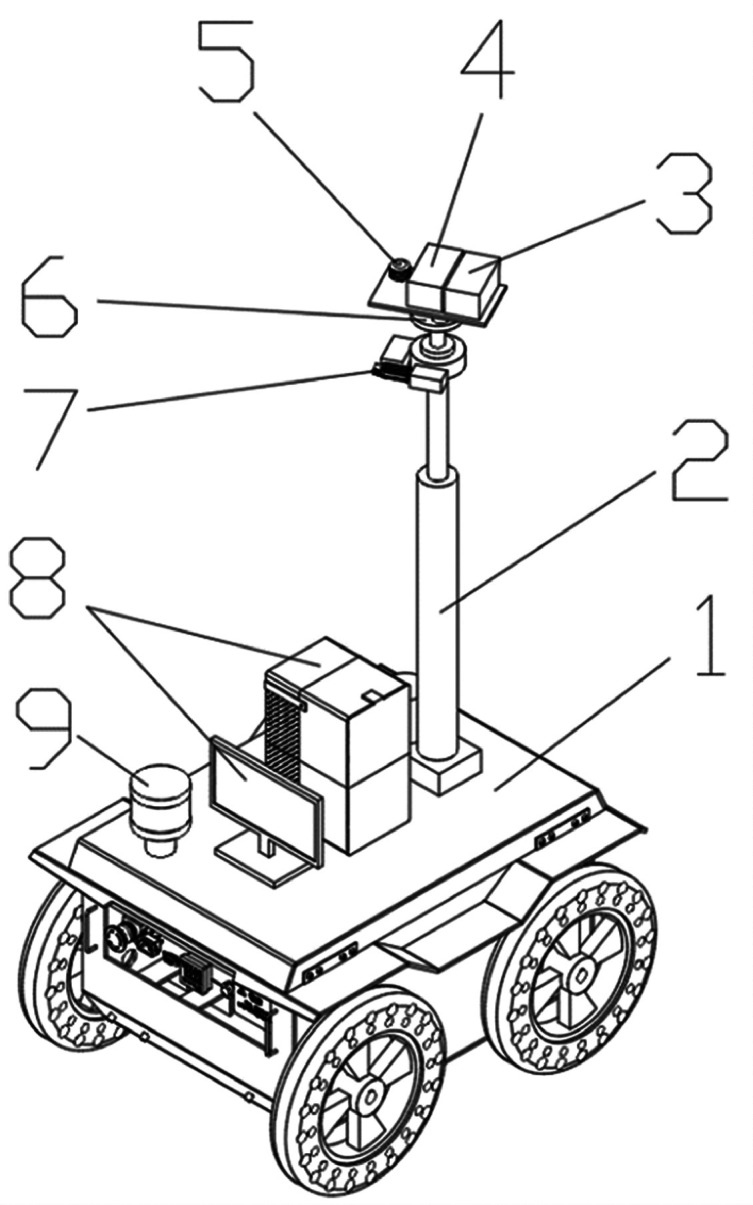
Poultry house inspection robot. 1: Inspection car; 2: Telescopic rod; 3: Infrard thermal imager; 4: Azure Kinect DK; 5: Environmental detector; 6: Platform swing mechanism; 7: Precision electric rotating platform; 8: Server; 9: Navigation radar.

The video data acquisition equipment used in this experiment is the poultry house inspection robot. The specific structure of the poultry house inspection robot is shown in Figure 1.

The infrared thermal imager (3), Azure Kinect DK (4), and Environmental detector (5) are fixed on the Telescopic rod (2), which is controlled by PLC, driven by servomotor, and fixed on the Inspection car (1). The Azure Kinect DK is a developer kit and PC external with a 1-megapixel advanced depth camera, 360° microphone array, 12-megapixel full HD camera, and orientation sensor for advanced computer vision and voice model development. It is less than 13 cm long and only 4 cm thick. Table 1 shows the detailed parameters of the device.

**Table 1:** Azure Kinect DK parameters.

Project	Parameters
Dimension	126.00 × 103.00 × 39.00mm
Sensor	Depth camera: 1-megapixel ToF GB camera: 12 megapixels, CMOS sensor with shutter IMU: 3D electronic accelerometer and 3D electronic gyroscope
Microphone	Seven-microphone circular array
I/O Interface	USB3.0 port Powered by USB-C or external power supply Synchronous I/O interface to synchronize with multiple devices
Compatible operating system	Windows 10 and Ubuntu18.04
System requirement	Windows 10 computers with 7th generation Intel CoreTM i3 processors (dual-core 2.4GHz with HD620 GPU or faster) Other features, such as body tracking, may require more advanced PC hardware
RAM	4GB

The inspection car (1) is based on SIMATIC S7-200 SMART PLC as the core and is equipped with a 16-wire Lidar, brushless DC motor, and encoder. The inspection car (1) is controlled by PLC and circulates in the chicken house under the action of LIDAR and navigation radar (9). The infrared thermal imager (3) is used to collect the thermal infrared images of chickens. Azure Kinect DK4 is used to capture RGB images of chickens. Telescopic rod (2) is used to adjust the height position of infrared thermal imager (3), Azure Kinect DK (4), environmental detector (5); The platform swing mechanism (6) and precision electric rotating platform (7) adjust the shooting angles of the infrared thermal imager (3), Azure Kinect DK (4), and environmental detector (5); The environmental detector (5) collects data on temperature, humidity, light intensity, and carbon dioxide concentration. The collected thermal infrared images, RGB images, temperature, humidity, light intensity, carbon dioxide concentration, and other data information are uploaded to server (8). Server (8) employs a data enhancement algorithm to screen the images and transmit them to the workstation.

#### Image Acquisition

In this study, image data were collected from the cage chicken breeding center affiliated with Shandong Agricultural University. The specific steps for image acquisition were as follows: Firstly, the AzureKinect sensor SDK on the server was configured, where the SDK included AzureKinect Viewer, AzureKinect Recorder, and AzureKinect Firmware Tool. Secondly, the navigation system was employed to establish the inspection route for the car to ensure that enough samples were collected. Then the server was connected with a USB-C cable and the lens angle was adjusted using the Azure Kinect viewer to ensure that the video content captured was complete. The frame count was set to 30 and the video resolution to 1920 × 1080p, each recording lasting 30 s, after the completion of the setting input instructions to start recording. The recordings featured light brown shell laying hens aged 18 to 25 wk within the cage door closed and within the cage door open. In order to obtain sufficient data set and satisfy sample diversity, multiple videos were recorded. The videos captured in two different states of the same cage were recorded in 1 group, and 4 groups were recorded in each cage. A total of 24 videos were recorded in six cages, of which 12 were with the coop open and 12 were with the coop closed. Finally, 21,600 images were extracted frame by frame. The dataset comprises 10,800 images with the cage door closed and an additional 10,800 images with the cage door open.

### Data Augmentation and Dataset Production

This paper employs 2 types of images: those with the cage door open and those with the cage door closed. This selection aims to ensure the accuracy of the image data and the clarity of the images used in network training. Out of the total 21,600 collected images, 13,884 images were selected due to their clear outlines and absence of significant noise.

Considering the pixel size of the collected images, which was 1,920 × 1,080p; their dimensions were excessively large for training purposes. An excessively large input could lead to model complexity, increasing the risk of overfitting. Furthermore, it could result in an overly large trained model, thereby reducing its applicability. Simultaneously, an excessively large image size occupies more GPU resources during the training process. This not only demands more from the hardware but also fails to ensure an improved trained model. Hence, prior to dataset creation, the size of 13, 884 images was initially adjusted to 720 × 405p using a Python program.

Within study, the processed images were divided into 4 different sets of files: Test A, Test B, Train A and Train B, where Train A and Test A were pictures of the cage door closed, and Train B and Test B were pictures of the cage door open. This division is established by randomly selecting images in the total dataset according to the ratio of 1:1:4:4 for Test A: Test B: Train A: Train B, thus forming a comprehensive dataset.

### Experimental Method

#### Generative Adversarial Networks

Generative Adversarial Networks (**GAN**) is a novel class of deep generative models that has recently gained significant attention (Saxena and Cao, 2021). Generative Adversarial Networks are machine-learning models composed of two neural networks: a Generator and a Discriminator. The fundamental concept behind GANs is to teach the network to produce lifelike data samples by placing generators and discriminators in opposition.

The generator aims to acquire the skill to generate novel samples resembling real data instances, while the discriminator's objective is to differentiate between genuine samples and generated ones. The generator takes random noise as input and produces synthetic samples. The discriminator categorizes the samples to determine whether they are real or generated. The generator and discriminator engage in iterative adversarial training, ultimately achieving the outcome wherein samples generated by the generator become indistinguishable from real ones.

The training process of GANs can be regarded as a zero-sum game process. Generators and discriminators engage in competitive training, iteratively adjusting parameters to enhance their respective capabilities. The generator's aim is to mislead the discriminator to the extent that it struggles to accurately ascertain sample authenticity. Conversely, the discriminator strives to meticulously differentiate between real and generated samples.

One of the primary strengths of GANs lies in their capacity to generate data that closely mirrors the distribution of the training data. This characteristic renders them exceptionally valuable in diverse applications, including but not limited to image generation, text generation, data augmentation, and anomaly detection. In the realm of image generation, GANs have found utility in crafting authentic images, including those of human faces, that find application in computer graphics and virtual reality domains.

#### Cyclic Consistent Migration Neural Network

The Cyclic Consistent Migration Neural Network (**CCMNN**) represents an unsupervised GANs network designed for image style conversion. By capturing the mapping relationships between different domains, it is possible to convert an image from one domain to another without the paired training data. Pix2Pix predates its emergence and aims at achieving image style conversion; however, it bears significant limitations. The primary challenge stems from the requirement that the two styles of images used for training need to be paired. In reality, acquiring a sufficient number of paired images exhibiting disparate styles yet identical content proves to be a daunting task. Consequently, this paper employs the CCMNN network model to eliminate the cage door from images featuring closed cage doors.

At the heart of CCMNN lies the concept of training through an adversarial network featuring two generators and 2 discriminators. One generator converts images from the source domain into the target domain, while the other generator transforms images from the target domain into the source domain. Two discriminators are employed to differentiate between the generated images and real images. The training process of CCMNN unfolds in 2 stages. During the initial stage, emphasis is placed on computing the adversarial loss. Through computation of the adversarial loss function, the network aims to diminish the disparity between the generated and real images. Additionally, it strives to amplify the discriminator's capacity to accurately distinguish between the two, thereby encouraging the generator to produce increasingly lifelike images.

**Figure 2:**
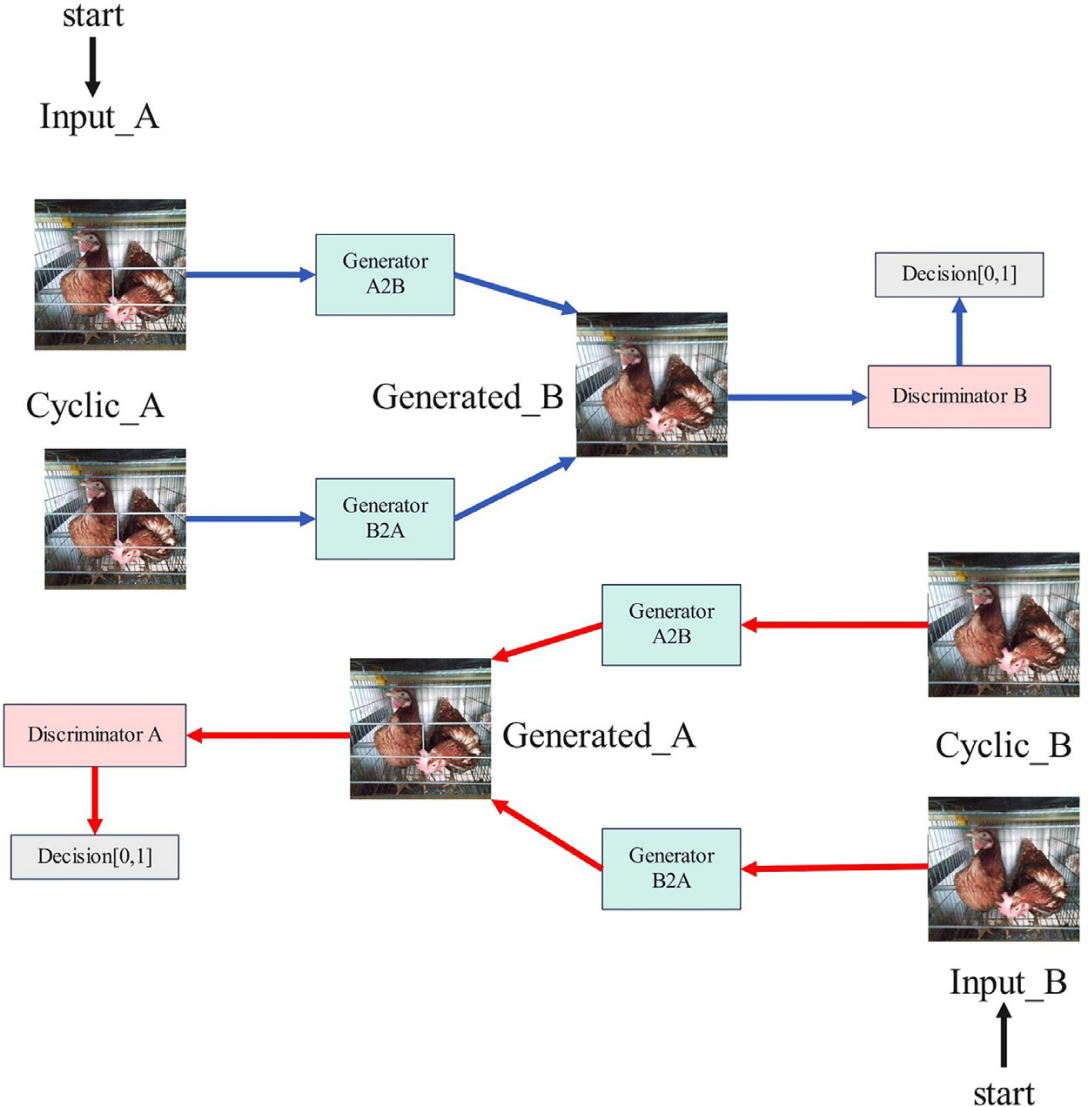
Network structure diagram of CCMNN.

**Figure 3:**
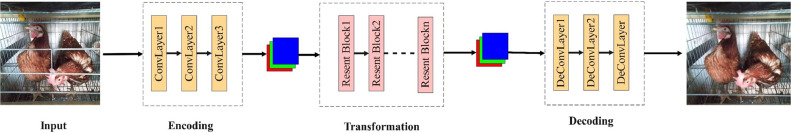
Generator.

**Figure 4:**
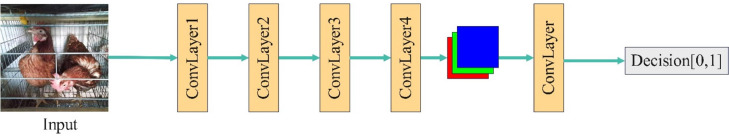
Discriminator.

In the second stage, focus shifts to the computation of cyclic consistency loss. Images from domain A are transformed into domain B images via the generatorA2B, and subsequently, these transformed images are reconverted to domain A images through the generatorB2A. Cyclic consistency loss is determined by evaluating the pixel-level disparity between the image reverted to domain A and the initial image within domain A. Likewise, a corresponding cyclic consistency loss is derived by conducting analogous operations on images from domain B. The cumulative cyclic consistency loss is then obtained by adding the two individual cyclic consistency losses. During the training process, constant optimization of both adversarial loss and cyclic consistency loss ensues, contributing to the enhancement of the CCMNN's capacity for image conversion and preservation of consistency. The network structure of CCMNN is shown in Figure 2. Figure 3 shows the generator in the CCMNN structure and Figure 4 shows the Discriminator in the CCMNN structure.

#### YOLOv8

Built upon the accomplishments of its predecessors, YOLOv8 stands as a cutting-edge model that introduces fresh features and enhancements to bolster performance and flexibility (Sharma et al., 2023). Notable innovations comprise a novel backbone network, an advanced Anchor-Free detection header, and a fresh loss function capable of operating across a range of hardware platforms, spanning CPUs to GPUs.

The Backbone and Neck segments of the backbone network supersede YOLOv8’s C3 structure with the C2f architecture, incorporating varying channel counts for diverse scale models. With the C2f structure featuring additional residual connections, facilitates a more copious gradient flow. Thus, the substitution of YOLOv8’s C3 structure with the C2f architecture equips YOLOv8 to harness a richer reservoir of gradient flow data, all while maintaining its lightweight nature.

The Head section brings forth two significant enhancements compared to YOLOv8. To start with, the architecture has transitioned from YOLOv8’s coupled Head to the contemporary mainstream Decoupled head structure (Decoupled Head), isolating the classification and detection components. Secondly, there has been a shift from Anchor-Based to an Anchor-Free paradigm. While prioritizing enhanced detection efficacy, the network structure has been streamlined.

When computing Loss, an adopted strategy is the task-aligned assigner positive sample matching, along with the incorporation of distribution focal loss (**DFL**). Positive samples are chosen by the Task-Aligned Assigner based on weighted scores derived from classification and regression scores, thereby training the network to prioritize dynamic attention and superior positive samples. Distribution focal loss aims to optimize the probability of the two positions nearest to the label using cross-entropy, expediting the network's ability to rapidly concentrate on the distribution of proximate regions aligned with the target location.

**Figure 5:**
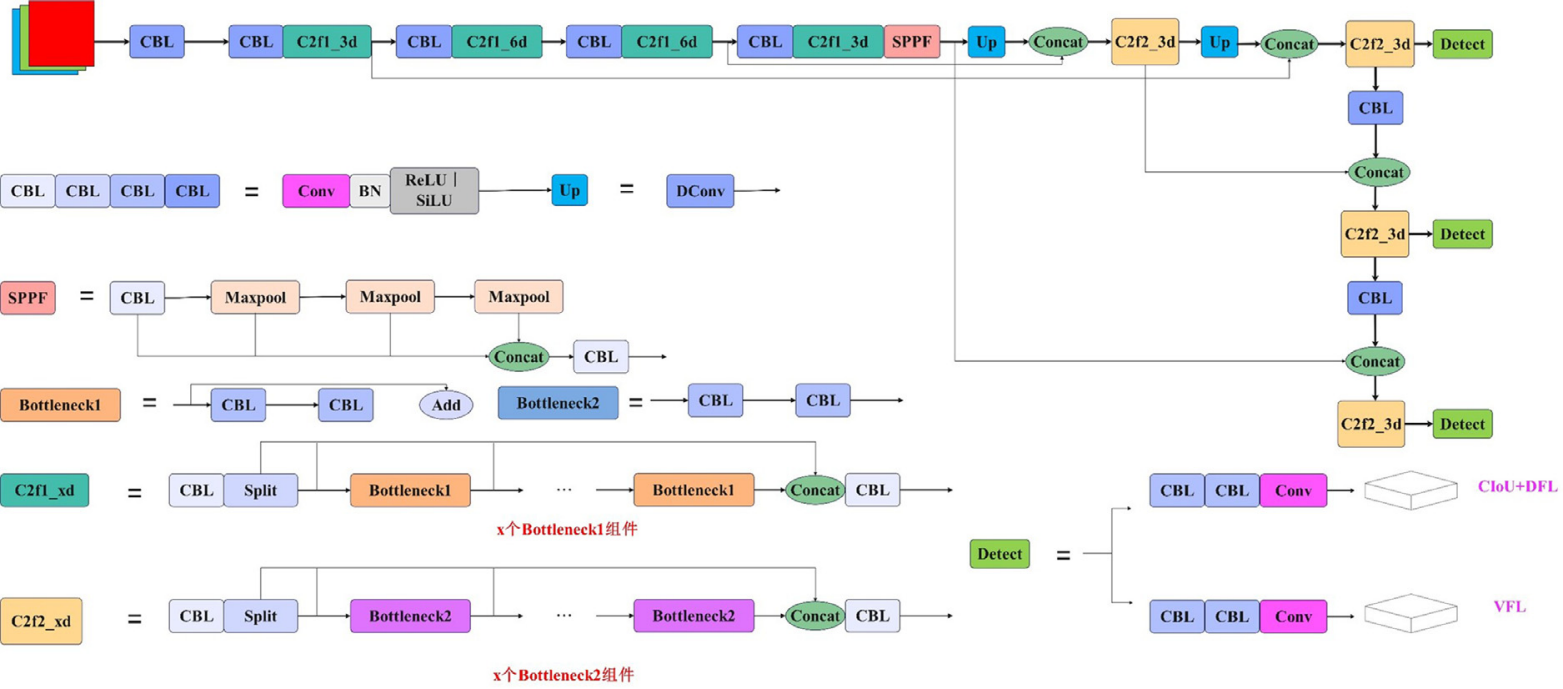
YOLOv8 network structure.

In the training part, the training method of YOLOX is adopted, and the Mosaic enhancement operation is turned off in the last 10 epochs, which can effectively improve the accuracy. The network structure of YOLOv8 is shown in Figure 5 below.

## Model Evaluation Index

### Image Quality Evaluation Index

The image quality evaluation criteria consist of peak signal-to-noise ratio (**PSNR**) and structural similarity index measure (**SSIM**). Peak signal-to-noise ratio is frequently employed to assess signal reconstruction quality in areas like image compression, and it is often simply defined by the mean square error (**MSE**). For 2 monochromatic images I and K of size m × n, when 1 approximates the noise of the other, their MSE is defined as follows:

(1) $$\text{MSE}=\frac{1}{\text{mn}}\sum_{i=0}^{m-1}\sum_{j=0}^{n-1}∥I(i,j)-K(i,j){∥}^{2}$$

PSNR is defined as:

(2) $$\text{PSNR}=10\cdot \log_{10}\left(\frac{{\text{MAX}}_{I}^{2}}{\text{MSE}}\right)$$

Where ${\text{MAX}}_{I}$ is the maximum pixel value of the image color. If each pixel is represented by 8-bit binary, then the value is 255.

SSIM is a full-reference image quality evaluation index. It measures the image similarity of sample *X* and sample *Y* from luminance, contrast and structure respectively. SSIM is defined as:

(3) SSIM(X,Y)=l(X,Y)⋅c(X,Y)⋅s(X,Y)

(4) $$c\left(X,Y\right)=\frac{2\sigma_{X}\sigma_{Y}+C_{2}}{\sigma_{X}^{2}+\sigma_{Y}^{2}+C_{2}}$$

(5) $$s\left(X,Y\right)=\frac{2\sigma_{\text{XY}}+C_{3}}{\sigma_{X}\sigma_{Y}+C_{3}}$$

(6) $$l\left(X,Y\right)=\frac{2\mu_{X}\mu_{Y}+C_{1}}{\mu_{X}^{2}+\mu_{Y}^{2}+C_{1}}$$

$$C_{1}={\left(k_{1}L\right)}^{2}$$

$$C_{2}={\left(k_{2}L\right)}^{2}$$

$$C_{3}=C_{2}/2$$

$$k_{1}=0.01\;k_{2}=0.03$$

Where *μ*_X_ is the mean value of *X, μ*_Y_ is the mean value of *Y*, $\sigma_{X}^{2}$ is the variance of *X,* $\sigma_{Y}^{2}$ is the variance of *Y*, σ_XY_ is the covariance of *X* and *Y, L* is the range of pixel values.

### Evaluation Criteria for Object Detection Models

How to judge the quality of a target detection model can be roughly evaluated from 3 aspects: Classification Accuracy can generally be measured by Accuracy, Precision, Recall Rate, PR curve, AP, mAP, etc. Positioning Accuracy can generally be evaluated by IoU; Running speed is generally measured by fps.

Accuracy represents the probability of correctly classifying all the samples; Precision signifies the number of true positive samples among all the recalled positive samples; Recall rate indicates the number of samples recalled; The PR curve is a graph plotted using Recall rate and Accuracy values; AP, standing for Average Precision, is a commonly used metric in retrieval and regression tasks. It is, in fact, equivalent to the area under the PR curve. mAP refers to the average AP across multiple categories (Everingham et al., 2014).

IoU stands for Intersection over Union, calculating the ratio of intersection to the union between the “predicted border” and the “true border” (Zheng et al., 2022). Fps, or Frames per second, refers to the rate at which images are processed per second during model evaluation. The various formulas are as follows:

(7) $$A=\frac{TP+TN}{P+N}$$

(8) $$P=\frac{TP}{TP+FP}$$

(9) $$R=\frac{TP}{TP+FN}$$

(10) $$\text{AP}=\frac{1}{101}\sum_{R\in \{0,0.01,...1\}}p_{\text{int}erp(R)}$$

(11) $$p_{\text{int}erp(R)}=\max_{R^{\prime }\geq R}p\left(R^{\prime }\right)$$

(12) $$mAP=\frac{1}{k}\sum_{i=1}^{k}AP_{i}$$

Where *A* stands for Accuracy, *P* stands for Precision. *TP* is True Positive example, indicating the quantity predicted as 1 and actually being 1; *FP* is False Positive example, indicating the quantity predicted as 1 and actually being 0; *FN* is False Negative example, indicating the quantity predicted as 0 and actually being 1. The correspondence between *TP, TN, FP*, and *FN* for different categories of detected targets is shown in Table 2 below.

**Table 2:** Classification for different categories.

Categories	Real situation	Predicated results	
		Comb	Eye
For comb	Comb	*TP*	*FN*
	Eye	*FP*	*TN*
For eye	Comb	*TN*	*FP*
	Eye	*FN*	*TP*

In Formula (11), *p^R’^* refers to the accuracy when the recall rate is *R’, p*_interp(R)_ namely is the maximum accuracy within the interpolation interval; in Formula (12), *k* = 2, is the number of detection categories, each representing the eye and the comb. *mAP*=mean Average Precision, is the average value of *AP* for each category.

## Experimental Design and Result Analysis

### Experimental Design

The experiment is based on the deep learning environment built by Ubuntu18.04.6LTS operating system, Python 3.7.0, Pytorch1.10.3, and CUDA11.3. The hardware environment used for training is Intel Xeon(R) Gold 5218R CPU. NVIDIA GeForce RTX 3090 GPU.

In this study, 13,884 images collected were initially divided into a training set and a test set, with a data split of 8:2, in which the ratio of images featuring the cage door open to those with the cage door closed was maintained at 1:1, and the CCMNN model was then trained using this dataset. Then the CCMNN model was used to convert 1, 388 images from the closed-cage state in the test set into the open-cage state. CCMNN parameter Settings are shown in Table 3.

**Table 3:** CCMNN parameters.

Project	Parameters
Epochs	200
netG	resent_9blocks
netD	basic
batch size	2
Model	cycle_GANs
Learning rate	0.0002

To assess the impact of the proposed algorithm on comb and chicken eye detection, a controlled experiment was devised. Initially, 3 distinct datasets were employed to individually train the YOLOv8 object detection algorithm. The first dataset-encompassed images featuring the cage door closed, the second contained images post-processing by the CCMNN network to remove the cage door, and the third encompassed images depicting the cage door open. The quantity of datasets employed across the 3 experiments remained constant, while the training set-to-test set ratio stood at 9:1. Table 4 shows the specific parameter Settings.

**Table 4:** Configuration of training parameters for each group.

Group	Training images	Number of images	Ratio of training set to test set
1	Images of cage door closed	1388	9:1
2	Images of cage door opening	1388	9:1
3	Images of cage door opening after processing	1388	9:1

In the training process of YOLOv8 model, varying parameters yield distinct outcomes. The value of batch size affects the training time. Larger batch sizes correspond to shorter training times per iteration. Considering the experimental setup, the batch size was configured at its maximum value of 32. The image size used in the experiment is 720 × 405 pixels. By opting for an input size of 720 × 720 pixels for the model, comprehensive image information can be captured, while simultaneously minimizing extraneous details like black borders generated during scaling and padding. The maximum training epoch correlates with both training time and effectiveness, and an epoch is the time required for all training sets to be trained once. A widely accepted criterion for model fitness is the convergence of the train loss and test loss curves, with no noticeable gap between them. Following numerous trials, it was deduced that setting the epoch value to 200 resulted in a plateaued loss curve. This choice prevented model overfitting and indicated the attainment of an optimal fit in the loss curve. Table 5 shows the specific parameter Settings.

**Table 5:** YOLOv8 parameter settings.

Project	Parameters
Epochs	200
Warmup	3.0
Patience	100
Batch size	16
Workers thread	8
Initial learning rate	0.01
Final learning rate	0.2
Weight decay	0.0005

### Experimental Results

**Figure 6:**
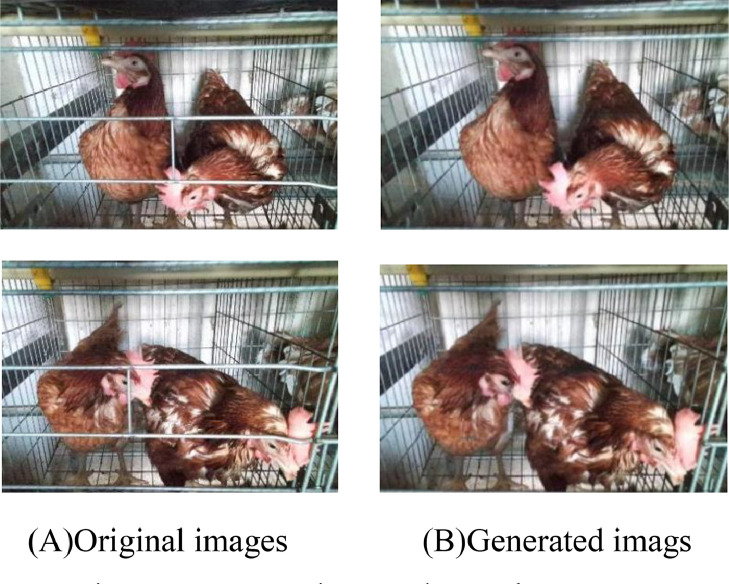
Conversion results on the test set.

The CCMNN network consists of two discriminators and 2 generators to ensure image conversion between different domains. The SSIM and PSNR of the trained CCMNN network model on the test set are 91.14% and 25.34dB, respectively. Figure 6 shows some of the image conversion results of the CCMNN network.

According to the conversion results of the test set, based on the powerful generation capability of the GANs network, CCMNN network can accurately generate realistic images with high definition that are very similar to the original images. For the obscured parts of the chicken's comb and chicken's eyes in the image, the CCMNN network can estimate the content of the obscured area through extensive training, utilizing surrounding pixel information through a lot of training, and fills these obscured areas while taking into consideration the image context and the characteristics of adjacent pixels. This process enhances the restoration of the obscured portions of the chicken's comb and chicken's eyes.

In this experiment, the verification of the improved detection effect of the CCMNN network was carried out through the design of 3 controlled experiment groups. In the first group of experiments, four target detection networks (YOLOv8s, YOLOv8n, YOLOv8m, and YOLOv8x) were trained using the dataset comprised of images with the cage door closed. The experimental design of the second group and the third group is the same as that of the first group, with the only difference being the dataset utilized. The dataset used in the second group is composed of images of the cage door opened after CCMNN network processing, and the dataset used in the third group is composed of images of the cage door opened.

For the convenience of discussion, when YOLOv8 is trained with images of cage door closed without CCMNN network processing, the trained network model is denoted as group UCI (unprocessed closed image); When YOLOv8 is trained with the image of the cage door opened after CCMNN network processing, the network model obtained is denoted as Group POI (processed open image); When YOLOv8 is trained with the cage door open image, the network model obtained by training is denoted as group UOI (unprocessed open image). The experimental results for each group on the verification set are shown in Table 6.

**Table 6:** Training results of YOLOv8 models in each group.

Class	Group	Evaluation index	Algorithms			
			YOLOv8s	YOLOv8n	YOLOv8m	YOLOv8x
All	UCI	Precision	0.839	0.842	0.835	0.837
		Recall	0.926	0.945	0.924	0.923
		mAP	0.936	0.930	0.921	0.921
	POI	Precision	0.941	0.953	0.942	0.941
		Recall	0.945	0.955	0.951	0.945
		mAP	0.966	0.956	0.944	0.956
	UOI	Precision	0.983	0.983	0.972	0.979
		Recall	0.966	0.979	0.978	0.982
		mAP	0.998	0.989	0.992	0.991
Comb	UCI	Precision	0.801	0.820	0.783	0.809
		Recall	0.913	0.938	0.928	0.928
		mAP	0.933	0.924	0.916	0.922
	POI	Precision	0.911	0.933	0.911	0.911
		Recall	0.932	0.941	0.946	0.943
		mAP	0.942	0.945	0.954	0.956
	UOI	Precision	0.966	0.988	0.955	0.966
		Recall	0.976	0.977	0.965	0.974
		mAP	0.989	0.992	0.989	0.987
Eye	UCI	Precision	0.878	0.864	0.887	0.864
		Recall	0.940	0.952	0.919	0.919
		mAP	0.939	0.937	0.925	0.921
	POI	Precision	0.902	0.944	0.942	0.954
		Recall	0.943	0.966	0.955	0.941
		mAP	0.954	0.942	0.938	0.967
	UOI	Precision	0.998	0.979	0.990	0.992
		Recall	0.971	0.982	0.991	0.991
		mAP	0.995	0.987	0.995	0.995

UCI:The data set consists of images of cage doors closed.

POI:The data set consists of images of cage doors opened obtained after processing by CCMNN.

UOI:The data set consists of images of cage doors opened obtained by taking.

In order to more intuitively show the detection effect of different models on different test sets, the 3 groups of models are tested on 2 test sets. One group of test sets consists of images of cage door closed, and the other group consists of images of cage door opened after CCMNN processing. The outcomes of UCI group's experiments on the test sets are shown in Table 7.

**Table 7:** Test results of group UCI models on test sets.

Test sets	Class	Evaluation index	Algorithms			
			YOLOv8s	YOLOv8n	YOLOv8m	YOLOv8x
Door closed images	All	Precision	0.836	0.842	0.836	0.833
		Recall	0.920	0.945	0.929	0.920
		mAP	0.932	0.930	0.919	0.920
	Comb	Precision	0.801	0.820	0.784	0.809
		Recall	0.913	0.938	0.938	0.928
		mAP	0.933	0.924	0.916	0.922
	Eye	Precision	0.870	0.864	0.887	0.857
		Recall	0.926	0.952	0.920	0.912
		mAP	0.930	0.937	0.923	0.918
Door opened images	All	Precision	0.876	0.920	0.892	0.844
		Recall	0.800	0.796	0.659	0.771
		mAP	0.892	0.905	0.783	0.863
	Comb	Precision	0.775	0.888	0.832	0.746
		Recall	0.690	0.713	0.571	0.676
		mAP	0.821	0.863	0.740	0.790
	Eye	Precision	0.978	0.951	0.953	0.941
		Recall	0.910	0.879	0.748	0.865
		mAP	0.964	0.947	0.826	0.936

When the group UCI of trained models was tested on the validation set, the overall precision were 0.839, 0.842, 0.835 and 0.837, and the recall rates were 0.926, 0.945, 0.924, and 0.923. In the test set composed of cage door closed images, the overall precision was 0.836, 0.842, 0.836, and 0.833, and the recall rates were 0.920, 0.945, 0.929, and 0.920. When tested on the test set composed of images of cage door opened generated after CCMNN network processing, the overall precision was 0.876, 0.920, 0.892, and 0.844, and the recall rates were 0.800, 0.796, 0.659, and 0.771. The outcomes of POI group's experiments on the test sets are shown in Table 8.

**Table 8:** Test results of group POI models on test sets.

Test sets	Class	Evaluation index	Algorithms			
			YOLOv8s	YOLOv8n	YOLOv8m	YOLOv8x
Door closed images	All	Precision	0.707	0.767	0.731	0.760
		Recall	0.886	0.783	0.819	0.842
		mAP	0.837	0.782	0.804	0.836
	Comb	Precision	0.673	0.682	0.618	0.658
		Recall	0.825	0.728	0.742	0.774
		mAP	0.795	0.726	0.738	0.778
	Eye	Precision	0.868	0.833	0.854	0.893
		Recall	0.911	0.838	0.896	0.810
		mAP	0.899	0.837	0.870	0.895
Door opened images	All	Precision	0.963	0.976	0.983	0.985
		Recall	0.839	0.968	0.979	0.844
		mAP	0.901	0.988	0.962	0.890
	Comb	Precision	0.941	0.984	0.955	0.961
		Recall	0.864	0.977	0.965	0.859
		mAP	0.912	0.969	0.989	0.978
	Eye	Precision	0.980	0.983	0.990	0.978
		Recall	0.831	0.987	0.991	0.855
		mAP	0.867	0.912	0.995	0.882

The precision of the group POI of trained models evaluated on the validation set is 0.941, 0.953, 0.942 and 0.941, and the recall is 0.945, 0.955, 0.951, and 0.945. After evaluation on the test set composed of cage door closed images, the overall precision of the model is 0.707, 0.767, 0.731, and 0.760, and the recall rate is 0.886, 0.783, 0.819, and 0.842. After evaluation on the test set composed of images of the cage door opened generated after CCMNN network processing the overall precision of the model is 0.963, 0.976, 0.983 and 0.985, and the recall rate is 0.839, 0.968, 0.979, and 0.844. The outcomes of UOI group's experiments on the test sets are shown in Table 9.

**Table 9:** Test results of group UOI models on test sets.

Test sets	Class	Evaluation Index	Algorithms			
			YOLOv8s	YOLOv8n	YOLOv8m	YOLOv8x
Door closed images	All	Precision	0.870	0.867	0.791	0.860
		Recall	0.896	0.883	0.839	0.842
		mAP	0.867	0.882	0.804	0.836
	Comb	Precision	0.873	0.782	0.618	0.768
		Recall	0.825	0.828	0.742	0.754
		mAP	0.795	0.826	0.738	0.768
	Eye	Precision	0.868	0.853	0.844	0.863
		Recall	0.906	0.938	.0896	0.820
		mAP	0.899	0.797	0.870	0.885
Door opened images	All	Precision	0.981	0.998	0.972	0.986
		Recall	0.848	0.988	0.978	0.834
		mAP	0.901	0.988	0.992	0.890
	Comb	Precision	0.961	0.984	0.955	0.961
		Recall	0.874	0.977	0.965	0.859
		mAP	0.924	0.991	0.989	0.909
	Eye	Precision	0.998	0.993	0.990	0.991
		Recall	0.822	0.992	0.991	0.829
		mAP	0.879	0.995	0.995	0.871

The evaluation data of the group UOI of trained models on the validation set showed that the overall precision of the model is 0.983, 0.983, 0.972, and 0.979, and the recall rate is 0.966, 0.979, 0.979, and 0.982. The evaluation data on the test set composed of cage door closed images showed that the overall precision of the model is 0.870, 0.867, 0.791, and 0.860, and the recall rate is 0.896, 0.883, 0.839, and 0.842. The evaluation data on the test set composed of images of the cage door opened generated after CCMNN network processing showed that the overall precision of the model is 0.981, 0.998, 0.972, and 0.986, and the recall rate is 0.848, 0.988, 0.978, and 0.834.

**Figure 7:**
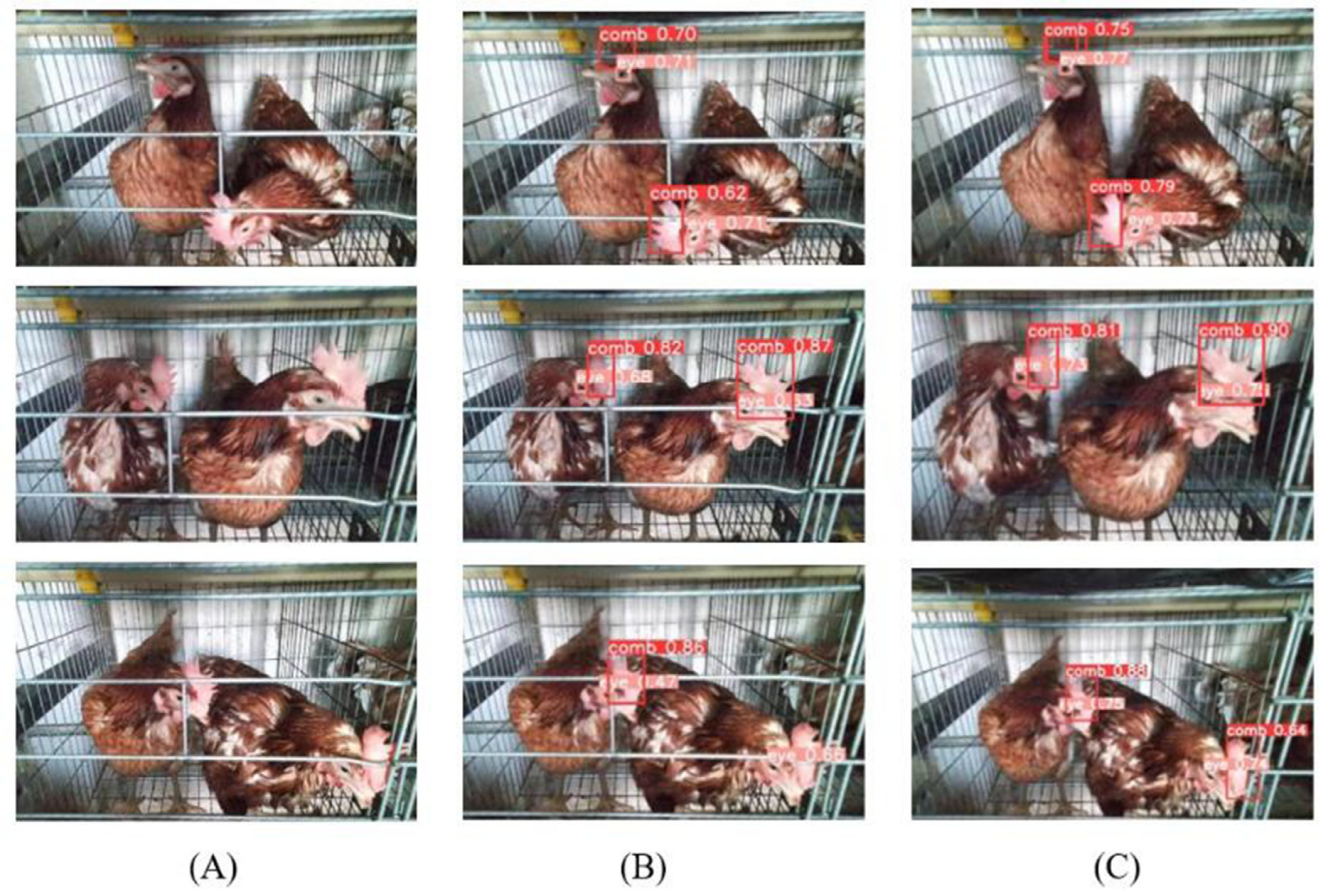
Object detection results. (A) Original images (B) Object detection results of the original images (C) Object detection results of the generated images.

The value of mAP in the test set is also counted in this paper. The mAP values of the first group of models on the test set composed of cage door closed images were 0.932, 0.930, 0.919, and 0.920, on the test set composed of cage door closed images. The mAP values of the test set with the cage door opened after CCMNN network processing were 0.892, 0.905, 0.783, and 0.863. The mAP values of the second group of models after the evaluation of the test set composed of the cage door closed images were 0.795, 0.726, 0.738, and 0.778, after the evaluation of the test set composed of the cage door closed images. The mAP values of the test set with the cage door opened after CCMNN were 0.901, 0.988, 0.962, and 0.890. The mAP values of the third group of models on the test set composed of cage door closed images were 0.867, 0.882, 0.804, and 0.836, on the test set composed of cage door closed images. The mAP values on the test set with the cage door opened after CCMNN network processing were 0.901, 0.988, 0.992, and 0.890. The performance of YOLOv8 target detection algorithm on the test set consisting of images processed by CCMNN network or not is shown in Figure 7.

### Analysis and Discussion

#### Analysis of Experimental Results

It can be seen from the table that compared with group UOI, the precision of the four network models YOLOv8s, YOLOv8n, YOLOv8m and YOLOv8x trained by group UCI decreased by 14.4, 14.1, 13.7, and 14.2%, and the recall rates decreased by 4, 3.4, 5.4, and 5.9%. Clearly, the precision and recall of each model in-group UCI on the validation set are markedly lower than those in-group UOI. Combined with the detection images generated by the models, it becomes evident that the primary reason is the incomplete capture of the shape and features of the comb and eyes of caged chickens by the target detection algorithm due to the presence of the cage door. The key feature points and texture information are challenging to extract and analyze using the detection algorithm. Consequently, the detection boxes of the models in-group UCI are unable to precisely align with the positions of the comb and the eyes of the chickens, leading to instances of missed and false detections. This discrepancy contributes to a lower detection performance in-group UCI compared to group UOI.

The precision and recall of each model in-group POI on the validation set are likewise inferior to those in group UOI. In comparison to group UOI, the overall precision of the four network models YOLOv8s, YOLOv8n, YOLOv8m, and YOLOv8x trained by group POI decreased by 4.2, 3, 3, and 5.6%, and the recall rates decreased by 2.1, 2.4, 2.7, and 3.7%. This indicates that while the CCMNN network eliminates the occlusion caused by the cage door on the comb and eyes of the chickens, and manages to recover the occluded area's contents to a certain degree, there may persist issues including information loss, suboptimal image quality, challenges in fully restoring intricate occlusions, and inadequacies in the post-occlusion removal processing by the CCMNN network after the occlusion is removed. These factors affect the performance of each model in-group POI, resulting in lower precision and recall rates compared to those in group UOI.

While the precision and recall rates of groups A and B are lower than those of group UOI, the precision and recall rates of each model in-group POI are notably superior to those in group UCI. In comparison to group UCI, the overall precision of the 4 network models YOLOv8s, YOLOv8n, YOLOv8m, and YOLOv8x achieved by group POI increased by 10.2, 11.1, 10.7, and 10.4%, and the recall rates increased by 1.9, 1, 2.7, and 2.2%. This is primarily due to the removal of the cage door in the image through CCMNN network processing, which restores the information of the obstructed portion covering the comb and chicken eyes, enhancing the completeness of these features. The elimination of the cage door liberates the target detection algorithm from occlusion interference, enabling it to focus more on the comb and chicken eyes. This improved visibility of key characteristics empowers the algorithm to capitalize on the restored information, resulting in more precise feature capture, and accurate comb and chicken eye identification and localization. The removal of the cage door makes the target detection algorithm no longer subject to the interference of occlusion, and focuses more on the comb and the chicken eyes, making full use of the recovered information to better capture important features, and more accurately distinguish and locate the comb and the chicken eyes.

The precision of each model in groups A, B, and C on the test set composed of images with the cage door opened after CCMNN network processing has been significantly improved compared to the precision on the test set composed of images without CCMNN network processing. The precision of group UCI has been improved by 4, 7.8, 5.6, and 1.1%. The precision of group POI has been improved by 25.6, 20.9, 25.2, and 22.5%. The precision of group UOI has been improved by 11.1, 13.1, 18.1, and 12.6%. Upon comparing the aforementioned 3 groups, it becomes evident that the CCMNN network processing has notably enhanced the detection performance of each model.

In summary, a cage-gate removal algorithm based on deep learning proposed in this paper utilizes the Cyclic Consistent Migration Neural Network (**CCMNN**), a network model based on consistent transfer neural networks, automatically eliminates the presence of cage doors that may affect the image's detection performance, while recovering occluded information and enhancing the overall integrity of the caged chicken's comb and eyes. As a result, the object detection algorithm is no longer hampered by occlusion, allowing it to effectively utilize the restored information for improved target identification, enhanced feature localization, and more accurate detection of the caged chicken's comb and eyes.

#### Compared With Existing Interference Removal Studies

In order to remove interference in images, and improve the detection effect, the researchers designed a variety of different methods. Sun et al. (2022) proposed an image enhancement approach for fish detection in complex underwater environments. This method can significantly improve detection accuracy. Ye et al. (2022) designed an end-to-end automatic pest detection framework based on a multi-scale attention-UNet (**MA-UNet**) model and monophasic images. Compared with the traditional model, the proposed model achieves a much better recall rate of 57.38% in detecting pest infested forest areas, while the recall rates of the Support Vector Machine (**SVM**), UNet, attention-UNet, and MedT models are 14.38, 49.33, 48.02, and 33.64%, respectively. The CCMNN network proposed in this paper as a cage door removal algorithm based on deep learning, achieves automatic elimination and restoration of some key information in the image, more complete outline images of caged chickens can be obtained using this algorithm, and the precision in detecting comb and eyes of caged chickens can be enhanced.

In previous studies, to mitigate the interference caused by the presence of cage doors and acquire comprehensive characteristic data of caged chickens, most researchers used the overhead view angle for detection and identification. However, the front-view camera can capture more details of caged chickens, which is more advantageous for the monitoring and identification of caged chickens. Therefore, in order to obtain better monitoring and identification effects and promote the development of monitoring technology, further research investment is essential in the monitoring and identification of frontal perspectives of caged chickens in future studies. The CCMNN network, proposed in this paper as a cage door removal algorithm based on deep learning, provides a reference for subsequent poultry researchers to deploy detection equipment, thereby ensuring that poultry detection systems can capture comprehensive front-facing image data, leading to enhanced accuracy in positioning and detection.

#### Discussion of Model Generalization Capability

**Figure 8:**
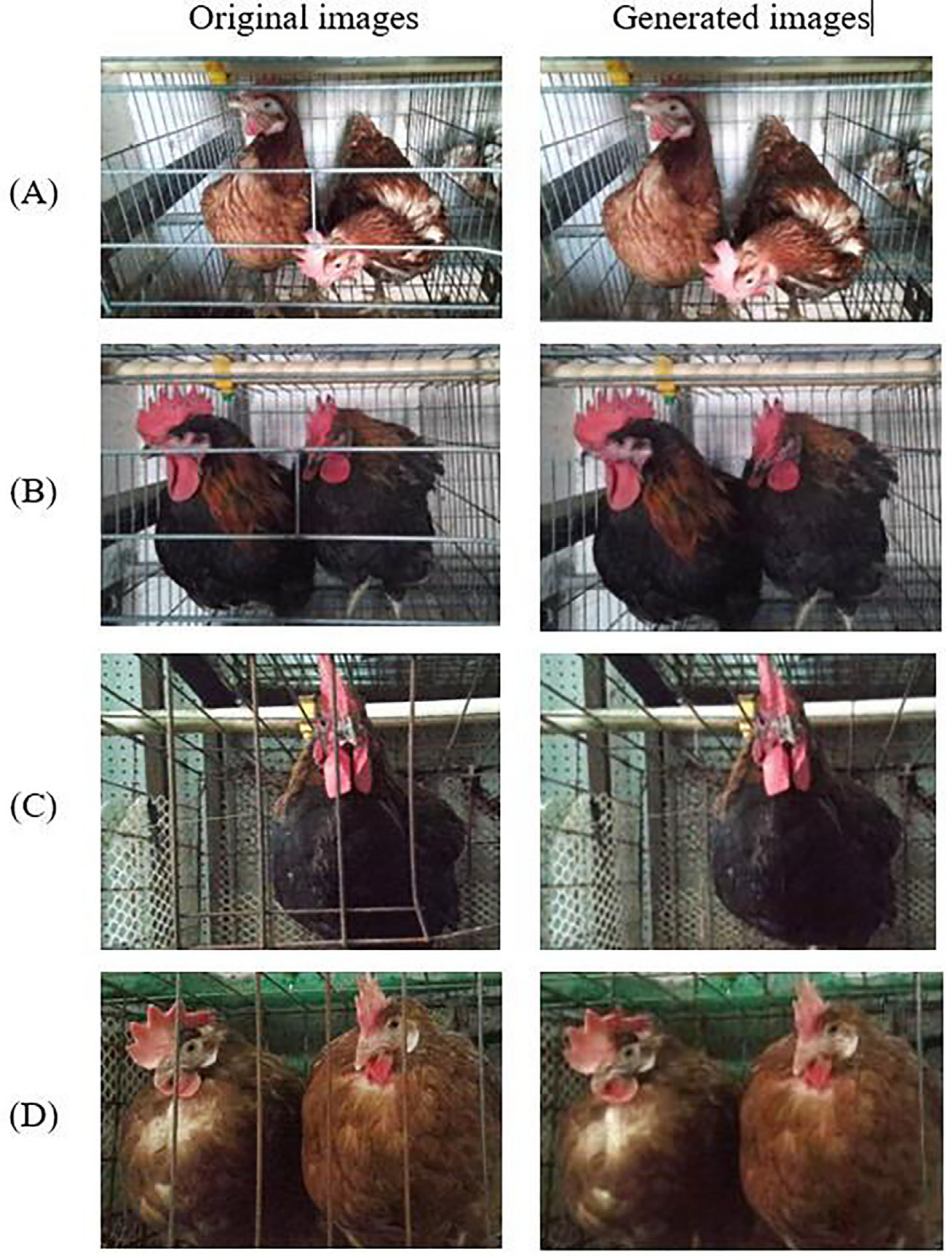
Conversion results on the different test sets.

The generalization capability of a model refers to the capability of the model to effectively adapt to unfamiliar data instances. An adequately generalizable model is characterized by its ability to effectively generalize its learned patterns and relationships to novel, previously unobserved data instances, rather than merely performing well on the training dataset. The generalization capability of a model is a pivotal metric for assessing the efficacy of a deep learning model. Hence, the examination of the generalization capability of the proposed CCMNN model is deliberated in this manuscript to gauge its viability for practical deployment. To achieve this aim, multiple validation experiments were conducted on various chicken breeds and different chicken cages. The specific test results are shown in the Figure 8.

In group A of Figure 8, the proposed CCMNN model effectively achieves precise image transformation while preserving intricate details of the original image by eliminating the cage door. Upon substituting HY-LINEVARIETYBROWN in group A with Gallus gallus domesticus in group B, the model demonstrates the capability to produce an image of the cage door opened while maintaining the intricate details of the original image without altering the cage structure. Comparing groups B and C, it is evident that the performance of the CCMNN model remains consistent regardless of the shift from horizontal cooperation to vertical cooperation within the same chicken species. Through a comparative analysis between group A and group D, it can be inferred that the conversion efficacy of the CCMNN model remains unaffected even in scenarios where there are alterations in the chicken type and chicken cage type. However, after comparing the performance of the model across different chicken breeds and various chicken coops, we conclude that the model exhibits outstanding generalization capability and broad applicability, making it effectively extendable to diverse chicken breeds and coop environments.

#### Limitation Analysis

In the detection of caged chickens, not only the cage door will block the characteristics of chickens, but also each chicken will block each other, because of which target detection algorithm cannot accurately identify the comb and eyes. In this study, only the influence of cage door was considered, and the mutual occlusion of chickens was not considered.

Therefore, in the subsequent study, a data enhancement algorithm was considered to expand the data set ensuring coverage of various chicken states. Improve the CCMNN algorithm, introduce more complex generator and discriminator structure, and add advanced neural network structure such as attention mechanism to better capture the subtle features in the image. Meanwhile, efforts were drawn to use the filling algorithm to estimate the content of the occluded part in order to recover it better. In addition to images, other modal information such as sound and video was also considered to be introduced. For the caged chicken scene, multi-modal information fusion can provide a richer feature representation for the model, which is conducive to the accuracy and fidelity of image conversion.

## CONCLUSIONS

In this paper, a deep learning-based cage-gate removal algorithm called the Cyclic Consistent Migration Neural Network (CCMNN) is proposed. This method enables the removal and recovery of specific information within the image. The experimental results show that the algorithm can accurately eliminate the cage door, and the occluded regions within the image can be effectively restored. Specifically, the detection precision of YOLOv8 can be enhanced by up to 11.1%, and the incidence of false detections and missed detections are significantly reduced. The proposed algorithm significantly enhances the detection performance of caged chickens, serves as a reference for future researchers in the poultry field for deploying detection equipment, which is conducive to accurate poultry production and farm monitoring.

## CRediT authorship contribution statement

**Zhenwei Yu:** Methodology, Writing – original draft. **Liqing Wan:** Methodology, Writing – original draft. **Khurram Yousaf:** Writing – review & editing. **Hai Lin:** Supervision, Validation. **Ji Zhang:** Data curation, Investigation. **Hongchao Jiao:** Data curation, Investigation. **Geqi Yan:** Resources, Visualization, Software. **Zhanhua Song:** Data curation, Investigation. **Fuyang Tian:** Writing – review & editing.
